# Targeted box and blocks test: Normative data and comparison to standard tests

**DOI:** 10.1371/journal.pone.0177965

**Published:** 2017-05-19

**Authors:** Kimberly Kontson, Ian Marcus, Barbara Myklebust, Eugene Civillico

**Affiliations:** 1 U.S. Food and Drug Administration, Center for Devices and Radiological Health, Office of Science and Engineering Labs, Division of Biomedical Physics, Silver Spring, Maryland, United States of America; 2 U.S. Food and Drug Administration, Center for Devices and Radiological Health, Office of Device Evaluation, Division of Neurological and Physical Medicine Devices, Silver Spring, Maryland, United States of America; Weill Cornell Medical College, UNITED STATES

## Abstract

**Background:**

The Box and Block Test (BBT) is a functional outcome measure that is commonly used across multiple clinical populations due to its benefits of ease and speed of implementation; reliable, objective measurement; and repetition of motion. In this study, we introduce a novel outcome measure called the targeted BBT that allows for the study of initiation, grasping, and transport of objects, and also of object release. These modifications to the existing test may increase the ecological validity of the measure while still retaining the previously stated benefits of the standard BBT.

**Methods:**

19 able-bodied subjects performed the targeted BBT and two other standard tests. Using an integrated movement analysis framework based on motion capture and ground force data, quantitative information about how subjects completed these tests were captured. Kinematic parameters at the wrist, elbow, shoulder, thorax, and head, as well as measures of postural control, were calculated and statistically compared across the three tests.

**Results:**

In general, the targeted BBT required significantly higher RoM at the elbow, shoulder, thorax and head when compared to standard tests. Peak angles at these joints were also higher during performance of the targeted BBT. Peak angles and RoM values for the targeted BBT were close to those found in studies of movements of able-bodied individuals performing activities of daily living.

**Conclusion:**

The targeted BBT allows analysis of repetitive movements, and may more closely model common real-world object manipulation scenarios in which a user is required to control a movement from pick-up to release.

## Introduction

Upper limb disability can result from a number of different conditions, including but not limited to stroke, musculoskeletal disorders, and amputation. For those persons undergoing treatment and/or rehabilitation for upper limb disabilities, functional outcome measures (OMs) are used to characterize the efficacy of a specific treatment or rehabilitation regimen. It is very common for clinicians to utilize several OMs that focus on performance of activities of daily living (ADLs), dexterity, or strength to provide adequate evaluation of functional abilities in a given clinical population. One of the most common measures used for such evaluation in the stroke population is the Box and Blocks Test (BBT) [1–3]. In addition to patients who have suffered stroke, the BBT has been utilized in a variety of other clinical populations such as those with multiple sclerosis[2], traumatic brain injury[2], fibromyalgia[4], and upper limb amputation [5–7], as well as the elderly [8]. In the BBT, one hundred and fifty 2.5cm^3^ wooden blocks in many different orientations are placed on the side of the partition with the testing hand. A subject’s score is equal to the number of these blocks transported over a 15.2-cm tall partition in one minute [9, 10]. The subject can select blocks in any order to transport over the partition as quickly as possible, with the only requirement being that the subject’s fingertips cross the vertical plane of the partition.

The standard BBT is a useful measure due to its ease and speed of implementation, reliable and objective measurement, and repetition of motion. However, there are several limitations associated with this test. As noted by Hebert et al.[5, 6], allowing the subject to choose which blocks to move results in tremendous variability in the trajectories employed, making comparisons among subjects difficult. Previous work [11] has also found that the joint angle ranges and peak angle values observed during the BBT are much lower than those that have been reported from able-bodied subjects performing typical ADLs such as perineal care, drinking from a cup, lifting objects from the ground and off shelves[12], and carton pouring [13]. While it remains difficult to develop a single task that will evaluate every aspect of functional performance, modifications to existing outcome measures may increase their ecological validity, while still maintaining the benefits of ease and speed of implementation; reliable, objective measurement; and repetition of motion.

**Fig 1:**
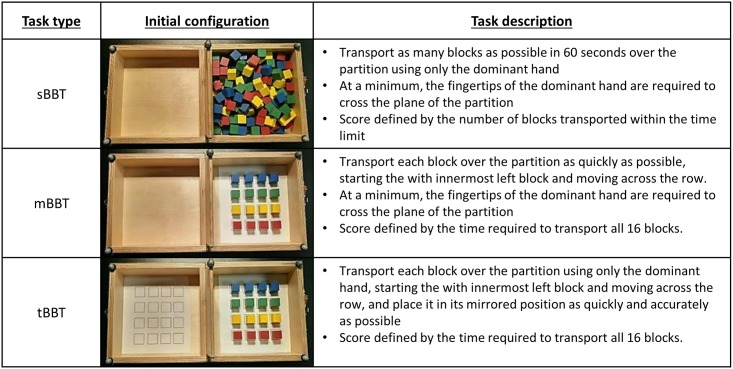
Description of tests.

In this study, we introduce a novel outcome measure (targeted Box and Blocks Test) that expands upon the instructions defined in the modified BBT introduced by Hebert et al [5, 6]. To create the modified BBT, which facilitates the comparison of trajectories and body movements, Hebert et al. standardized the placement and order of block pick-up [5, 6]. The same instructions in the standard BBT applied for the transport of the blocks in the modified BBT; subjects were instructed to drop the blocks on the other side of the partition, making sure that the fingertips of the tested hand crossed the partition plane. In order to assess control over the entire movement, we have introduced an additional instruction that requires the user accurately place each block in a specific position on the opposite side of the partition. Sheets of paper on which were printed identical 4x4 grids were placed inside of the box on each side of the partition (see Fig 1), giving participants a target within which to place the blocks. This allowed quantitative study of initiation, grasping, and transport of objects, and also of object release [14]. The targeted Box and Blocks Test (tBBT) still allows analysis of repetitive movements, but may more closely model common real-world object manipulation scenarios in which a user is required to control a movement from pick-up to release.

Several studies have shown the utility of motion capture in quantifying movement of the upper limb [5, 6, 12, 13, 15–21]. There are also studies that have employed the use of ground force plates to analyze the postural control of individuals performing standing tasks [22, 23]. Using an integrated movement analysis framework previously reported [11], the goals of the current study were to collect initial normative performance and movement data for the tBBT, and compare the results with the standard BBT (sBBT) and Hebert et al.’s modified BBT (mBBT).

## Methods

### Participants

For the current study, 19 right-handed subjects (10 female, 9 male; mean age 29.9 ± 8.3 years) with no upper limb disability were asked to perform the functional tasks. The study was approved by the Institutional Review Board (Research Involving Human Subjects Committee) of the U.S. Food and Drug Administration (RIHSC #14-086R). All subjects provided written informed consent prior to participating in the study.

### Functional tests

Participants performed both the modified BBT (mBBT) and the targeted BBT (tBBT). In a previous study, we reported the movement data for the same subjects performing the standard BBT (sBBT) [11]. These data are shown here for comparison. All subjects were right hand dominant, and only performed the tests with the right hand. Fig 1 gives a brief description of each functional test, along with a photograph of the box and blocks in their initial state. Subjects were asked to start with both hands resting on the table.

### Data collection and analysis

The integrated movement analysis framework previously described in [11] was used to collect kinematic and kinetic data during performance of the BBTs. An eight- camera Vicon^™^ motion capture system (Vicon, Oxford, UK) was synchronized with two Kistler ground force plates (Kistler, Winterthur, Switzerland). Data were collected from the optical cameras and ground force plates at a rate of 150 Hz and 900 Hz, respectively. A digital video camera was also incorporated into the framework to capture frontal recordings of the subjects performing each test.

Reflective markers were placed on the subject’s head, torso, arms, and pelvis according to the Plug-In-Gait upper body model from Vicon^™^. Measurements of height, weight, hand/wrist/elbow width and shoulder offset were taken and used for model calibration. To aid in data segmentation, five markers were also placed on the box: one on each corner and one on the partition. Subjects were asked to stand on the two ground reaction force plates, with one foot on each plate. The height-adjustable table was positioned such that the right anterior superior iliac spine marker was 10 cm below the surface of the table. The BBT apparatus was positioned 4 cm from the edge of the table, with the center partition aligned with the subject’s midline.

Joint angle trajectories were calculated using YXZ Euler angle decomposition. Thorax and head angles were determined by how the thorax and head moved relative to the global coordinate system. Wrist, elbow, and shoulder angles were determined relative to defined body segments. Details on the calculation of kinematic parameters can be found in [24]. Kinematic data were filtered with a 4^th^ order, zero lag, lowpass Butterworth filter at 6 Hz. Each test was segmented into several trials, where trial start was defined as the initiation of the approach to pick up a block, and trial end was defined as the release of the block. The position of the right finger marker, placed on the second metacarpal, relative to the markers placed on the box aided in the segmentation of each trial. Trials were then averaged and compared across tests for several kinematic parameters: wrist flexion (-) and extension (+); forearm internal rotation; elbow flexion; shoulder abduction and flexion; torso forward flexion (+) and torso left bending (+); head flexion (+)/extension (-), and left tilt (+). Total range of motion (RoM) and maximum angle for each kinematic parameter were calculated for each subject performing all BBTs, and averaged.

The force plate data were downsampled to 150 Hz and filtered using a 4^th^ order, zero lag, low pass Butterworth filter at 10 Hz, and segmented for each subject. Since the mBBT and tBBT require transport of 16 blocks while the sBBT requires transport of as many blocks as possible in one minute (i.e. > 16 blocks), CoP parameters derived from subjects performing mBBT and tBBT cannot be compared with sBBT. To provide an equal comparison of the CoP parameters, the force plate data from the median 16 blocks transported during the sBBT were used. The tri-axial forces and moments were then transformed into planar CoP trajectories in the XY plane defined by anterior-posterior (AP) and medial-lateral (ML) axes. These CoP trajectories were used to calculate quantitative metrics such as CoP area and orientation [23, 25], ML range, AP range, velocity, acceleration, and distance traveled [26, 27]. Due to technical errors, force plate data from three subjects were not captured. All data processing was completed using MATLAB (Mathworks, Inc.; Natick, MA; USA, version R2014b)

### Statistical analysis

The differences between the calculated kinematic and kinetic parameters for the sBBT, mBBT and tBBT were statistically evaluated using the Friedman test. Each parameter was considered a discrete data set to which the Friedman Test was applied. Only a single statistical test was applied to each discrete data set; thus, there was no need to adjust for multiple comparisons. This non-parametric test was used due to the potential violation of the normality assumption for other parametric tests. Differences were considered significant at a level of p < 0.05. For those parameters that showed statistically significant results across the different BBTs, a Tukey post-hoc analysis at significance level of α = 0.05 was performed to determine which specific tests differed.

## Results

### Performance analysis

**Fig 2:**
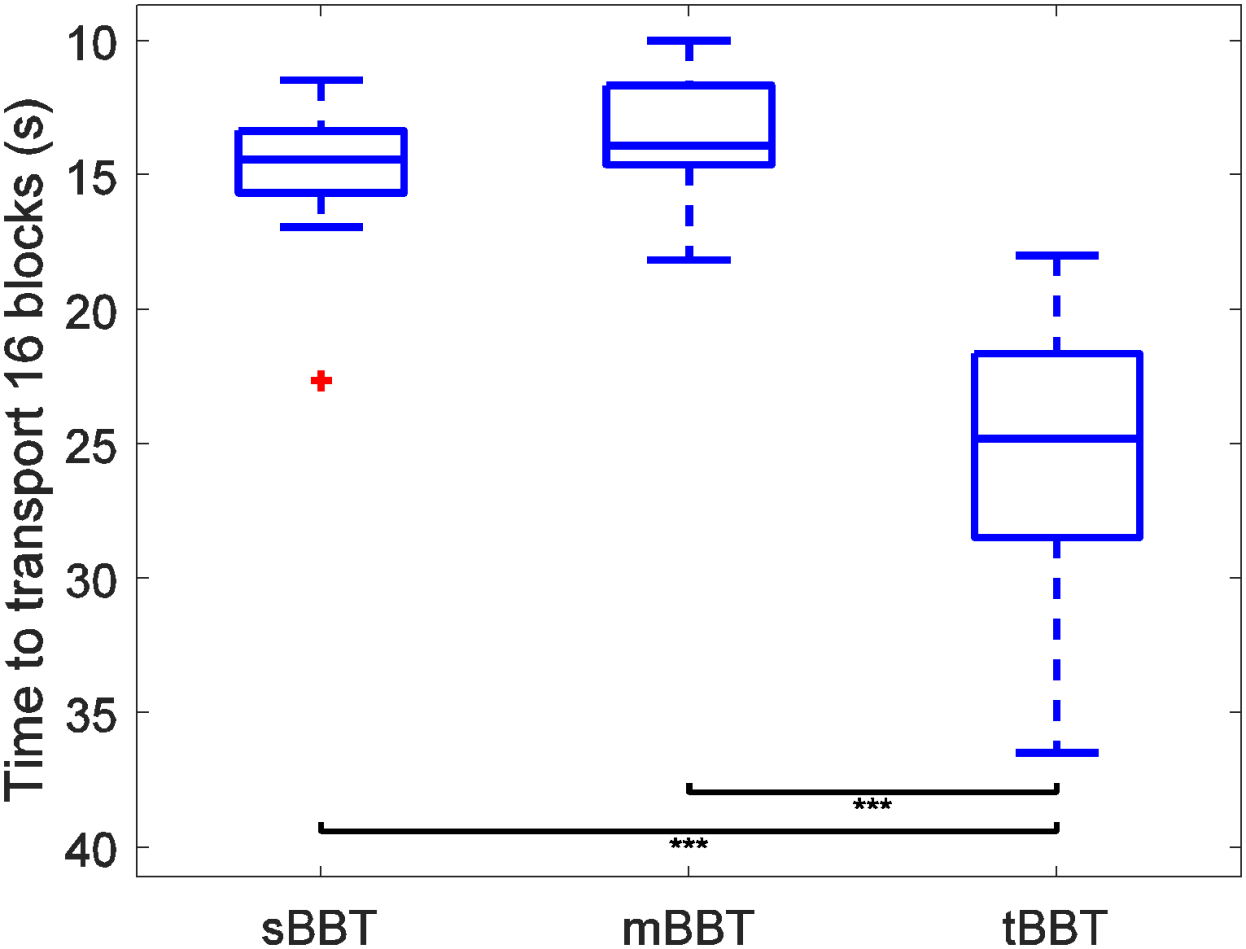
Distribution of subject scores for the sBBT, mBBT, and tBBT. Outliers are plotted as red crosses.

The distributions of scores for all subjects for each of the three BBT variants are plotted in Fig 2. To allow the sBBT to be compared to the 16-block variants, the time required to transport the first 16 blocks of the sBBT was calculated and used for comparison. In the following figures, one star indicates significance of p < 0.05; two stars, p < 0.01; and three stars, p < 0.001.

The distribution of scores for the tBBT was significantly different from the sBBT and mBBT scores, indicating that the tBBT produces different performance outcomes. No significant difference in performance between sBBT and mBBT was determined. Variability of the score distributions was also larger for the tBBT: the standard deviations of scores were 2.45 s, 2.15 s, and 5.14 s for the sBBT, mBBT, and tBBT, respectively.

### Kinematic data

Several kinematic parameters were analyzed to determine normal kinematics of movement when subjects performed the novel tBBT metric, and to compare the movement trajectories across the BBT variants. The following figures show the trajectories of each kinematic parameter as a function of percentage trial completion and the RoM for each kinematic parameter, averaged over all subject trials for a particular test. Raw kinematic data can be found in the S1 File.

#### Wrist angles

**Fig 3:**
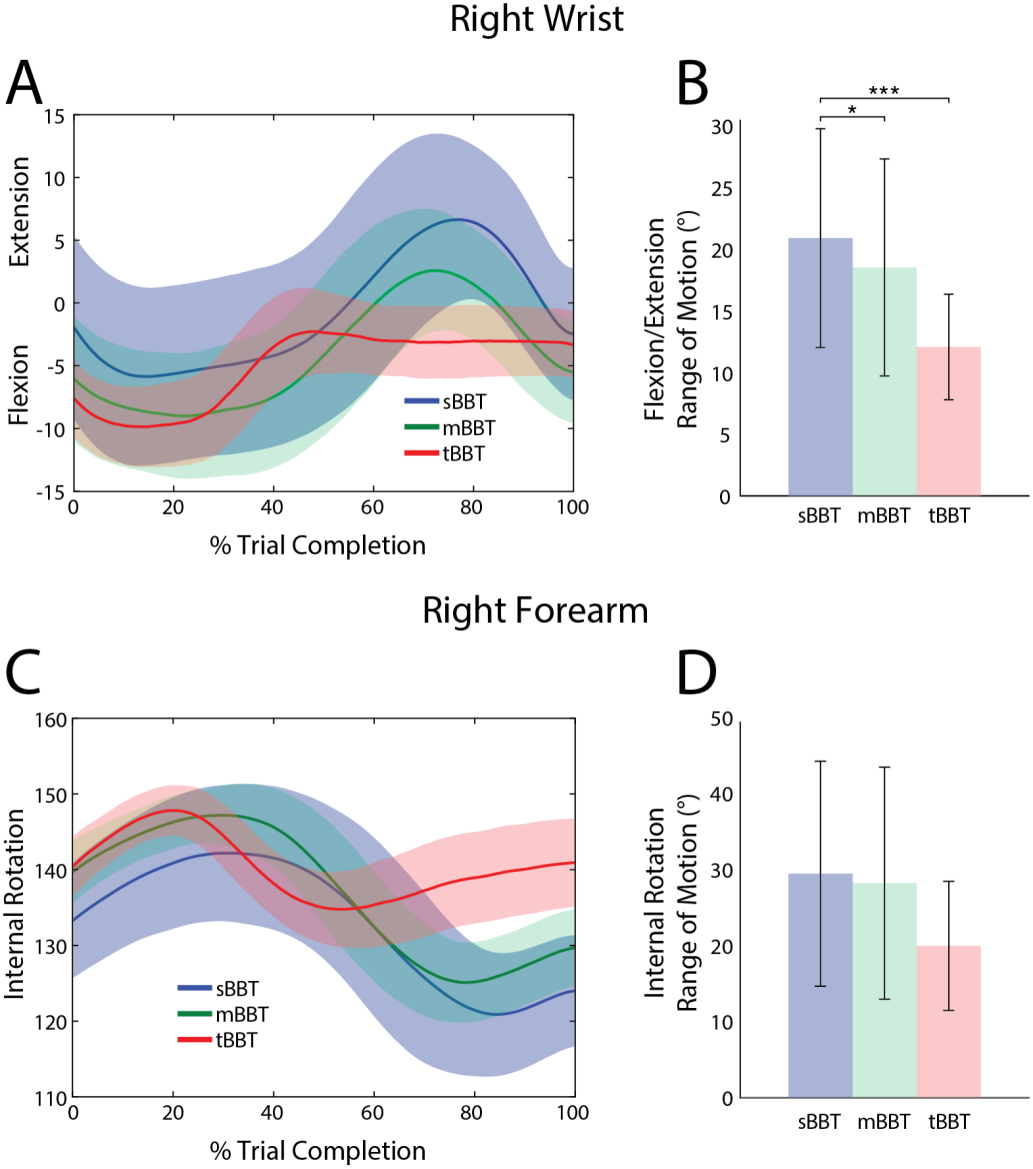
Kinematic trajectories and ROM of the wrist/forearm. Kinematic trajectories and ROM of the wrist/forearm for the sBBT (blue), mBBT (green), and tBBT (red). (A)Wrist flexion/extension and (C) forearm internal rotation trajectories for each test. (B) Wrist flexion/extension RoM and (D) forearm internal rotation RoM for each test.

Fig 3 shows the kinematic trajectories and ROM for right wrist extension/flexion and forearm internal rotation for all three BBTs. As subjects moved their right hand into the space below the box partition to pick up a block (i.e. 0% – 30% trial completion), wrist flexion increased (Fig 3A). For the sBBT and mBBT, the wrist gradually extended during block movement, peaking around 80% trial completion, before flexing again as the block was released. For the tBBT, however, wrist extension peaked shortly after block pick-up and remained constant until the block was placed on the opposite side of the partition. Significant differences were found between BBTs for the RoM of wrist flexion/extension. Tukey post-hoc comparisons showed that the wrist extension/flexion RoM was significantly greater during performance of the sBBT (p < 0.05) (Fig 3B). No significant differences were found in wrist flexion/extension RoM between the mBBT and tBBT.

Internal rotation of the forearm for all BBTs is shown in Fig 3C. If the hand were lying flat on a table, an angle of 0° corresponds to palm facing up, and an angle of 180° corresponds to palm facing down. On average, internal rotation of the forearm was approximately 137° at trial start for all BBTs. As the hand approached a block, internal rotation increased to a maximum angle of 145°, 149°, and 150° for the sBBT, mBBT, and tBBT, respectively. During block transport in the sBBT and mBBT, internal rotation then decreased to a minimum angle of approximately 121° and 125°, respectively, before the block was released. Forearm internal rotation ROM for the sBBT and mBBT were very similar (Fig 3D). For the tBBT, however, the RoM was about 10° less; this is not statistically different.

#### Elbow angles

**Fig 4:**
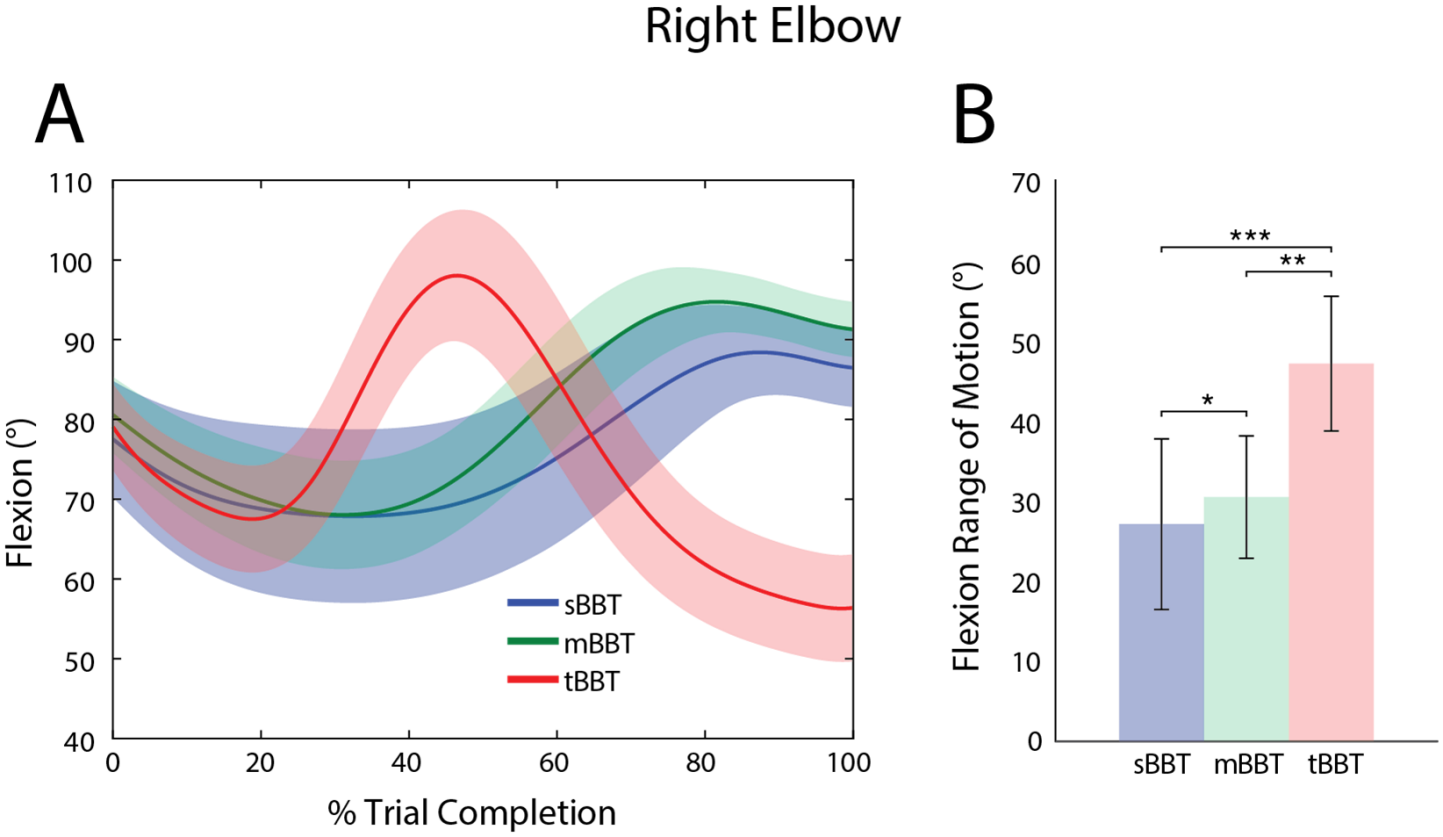
Kinematic trajectories and RoM of the right elbow. Kinematic trajectories and RoM of the right elbow for the sBBT (blue), mBBT (green), and tBBT (red). (A) Right elbow flexion as a function of percent trial completion, averaged over all subjects for each BBT. (B) Right Elbow RoM

Elbow flexion is shown in Fig 4A as a function of percent trial completion. For the sBBT and mBBT, the same general movement patterns were observed: the subjects started to extend the elbow as they approached a block, and flexed to a maximum angle just before releasing the block to the other side of the partition. The same initial elbow movement was seen in subjects performing the tBBT, but the peak elbow flexion occurred much earlier in the trial and was followed by extension of the elbow to control the placement and release of the block.

Significant differences (p < 0.05) between BBT variants were also observed for elbow RoM. Tukey post-hoc comparisons indicate that all variants significantly differed from each other, with the highest elbow ROM in the tBBT and the lowest elbow RoM in the sBBT.

#### Shoulder angles

**Fig 5:**
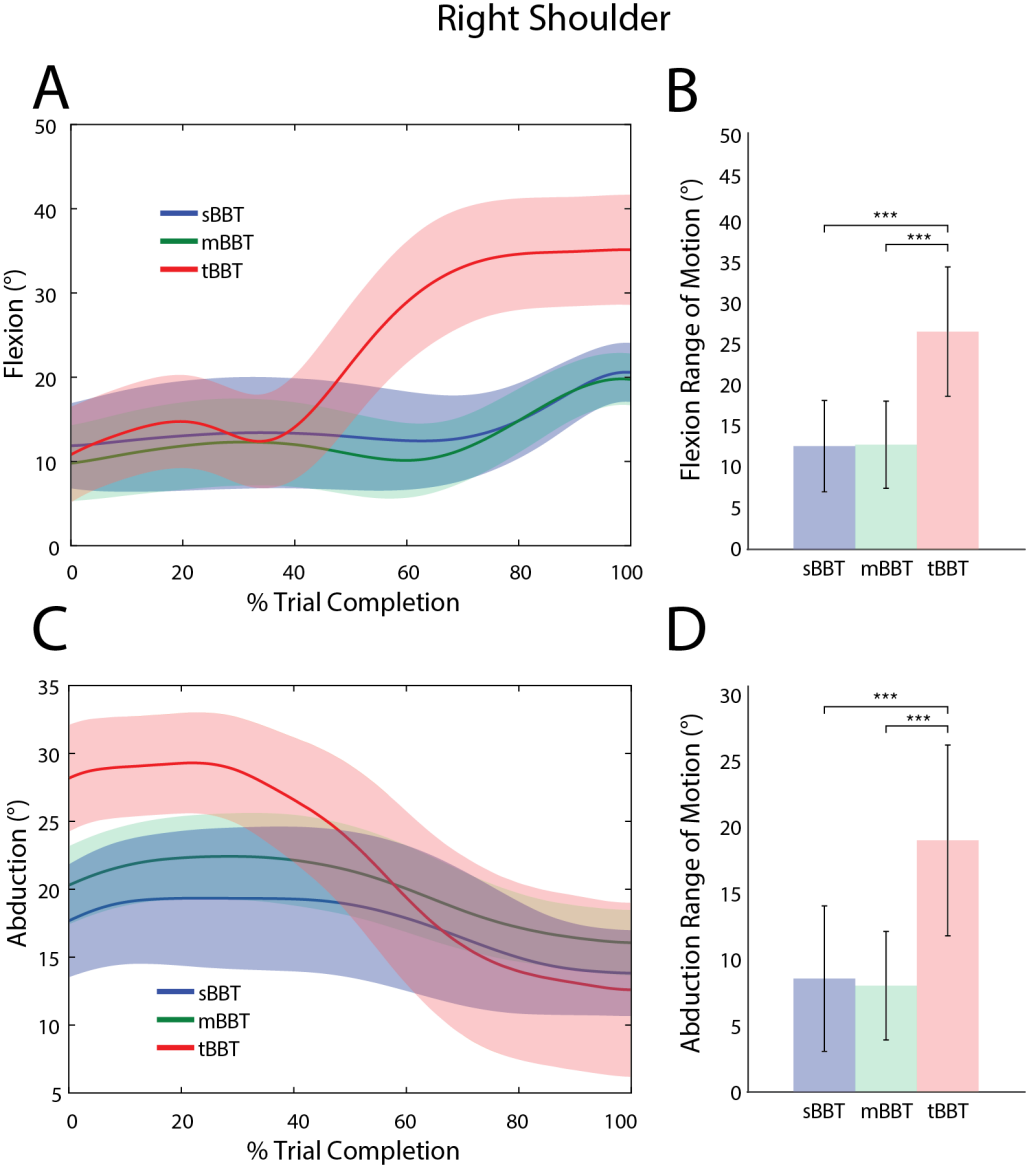
Kinematic trajectories and RoM of the right shoulder. Kinematic trajectories and RoM of the right shoulder for the sBBT (blue), mBBT (green), and tBBT (red). (A)Shoulder flexion and (C) shoulder abduction trajectories for each test. (B) Shoulder flexion RoM and (D) shoulder abduction RoM for each test.

Shoulder flexion and abduction trajectories, as well as RoM are shown in Fig 5 for the different tests. At the start of each trial, the shoulder flexion angle was, on average, about 10° for all three tests (Fig 5A). During transport of each block, the tBBT required significantly more RoM to successfully move the block over the partition and place it on the opposite side (Fig 5B). There was no significant difference in the shoulder flexion RoM between the sBBT and mBBT.

Shoulder abduction and flexion RoM values significantly differed between BBT tasks (p < 0.05). As seen in Fig 5, and through Tukey post-hoc comparisons, the tBBT shoulder RoM values were significantly greater than sBBT and mBBT.

The shoulder abduction RoM in the sBBT and mBBT were both significantly less than the RoM required to perform the tBBT (Fig 5D). At trial start, the shoulder was abducted about 10° more in the tBBT than sBBT and mBBT (Fig 5C). For all tests, subjects generally ended each block transport with a shoulder abduction angle between 13° – 17° (Fig 5C).

#### Thorax angles

**Fig 6:**
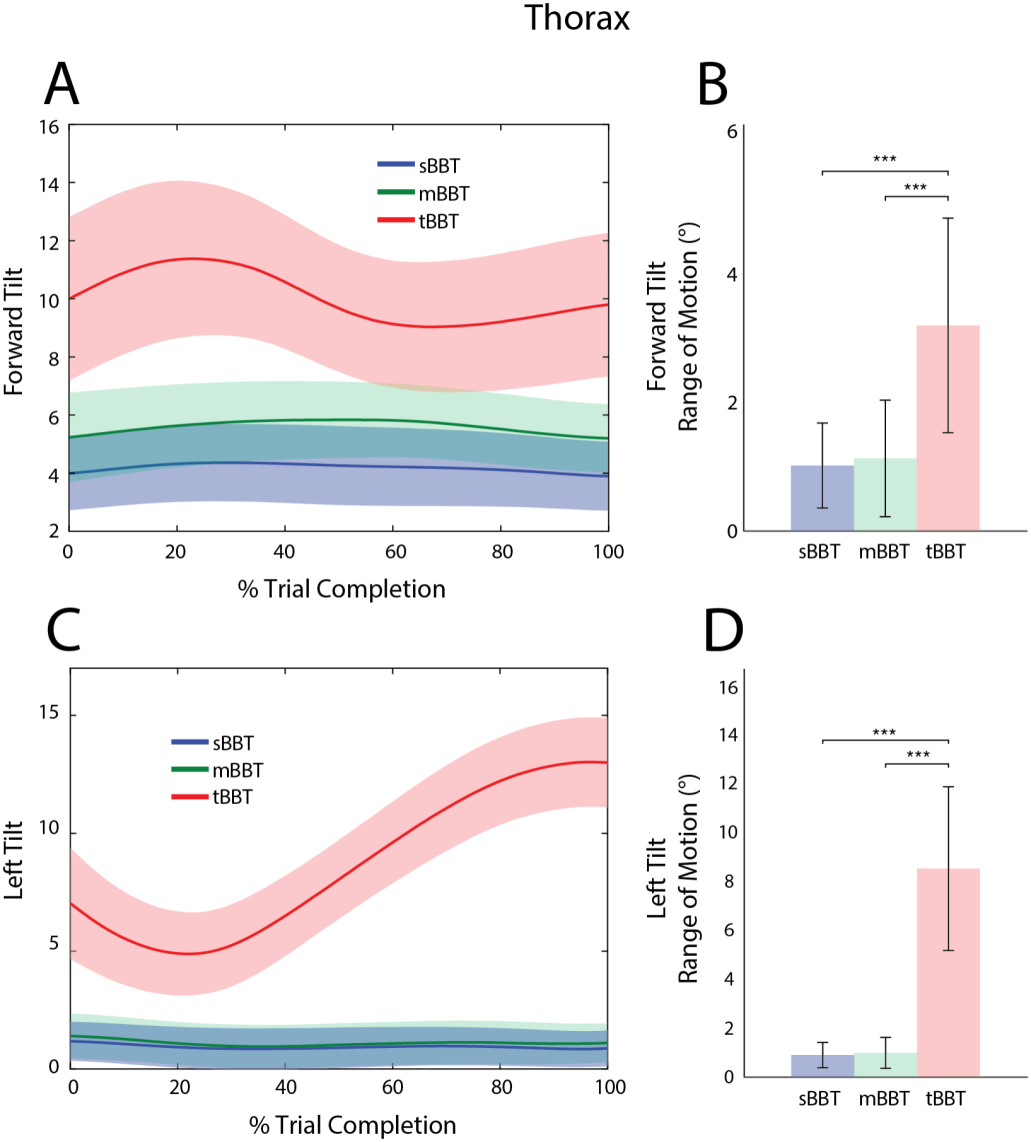
Kinematic trajectories and RoM of the thorax. Kinematic trajectories and RoM of the thorax for the sBBT (blue), mBBT (green), and tBBT (red). (A) Forward tilt and (C) left tilt trajectories for each test. (B) Forward tilt RoM and (D) left tilt RoM for each test.

Average thorax flexion (forward tilt) and lateral bending (left tilt) trajectories and RoM are shown for all subjects (Fig 6). In the sBBT and mBBT, forward tilt and left tilt trajectories did not significantly differ in the required RoM or peak angle value, as subjects generally maintained an upright stance during task performance. In contrast, the tBBT elicited significantly different movements. At trial start, thorax forward tilt was approximately 10° during the tBBT compared to 4° and 5° for the sBBT and mBBT, respectively. During the block approach (0–25% trial completion), the tBBT forward tilt angle increased compared to the other tests, with a peak angle occurring at approximately 22% of the total trial time (Fig 6A). This angle subsequently decreased as the subject transported the block over the partition, while still maintaining more flexion of the thorax compared to the sBBT and mBBT.

During block pick-up, subjects started with a greater left tilt angle in the tBBT task compared to the sBBT and mBBT (Fig 6C) and had to significantly increase the RoM in order to complete the task (Fig 6D). The lateral position of the torso remained fairly upright near 1° throughout the entire trial for the sBBT and mBBT.

#### Head angles

**Fig 7:**
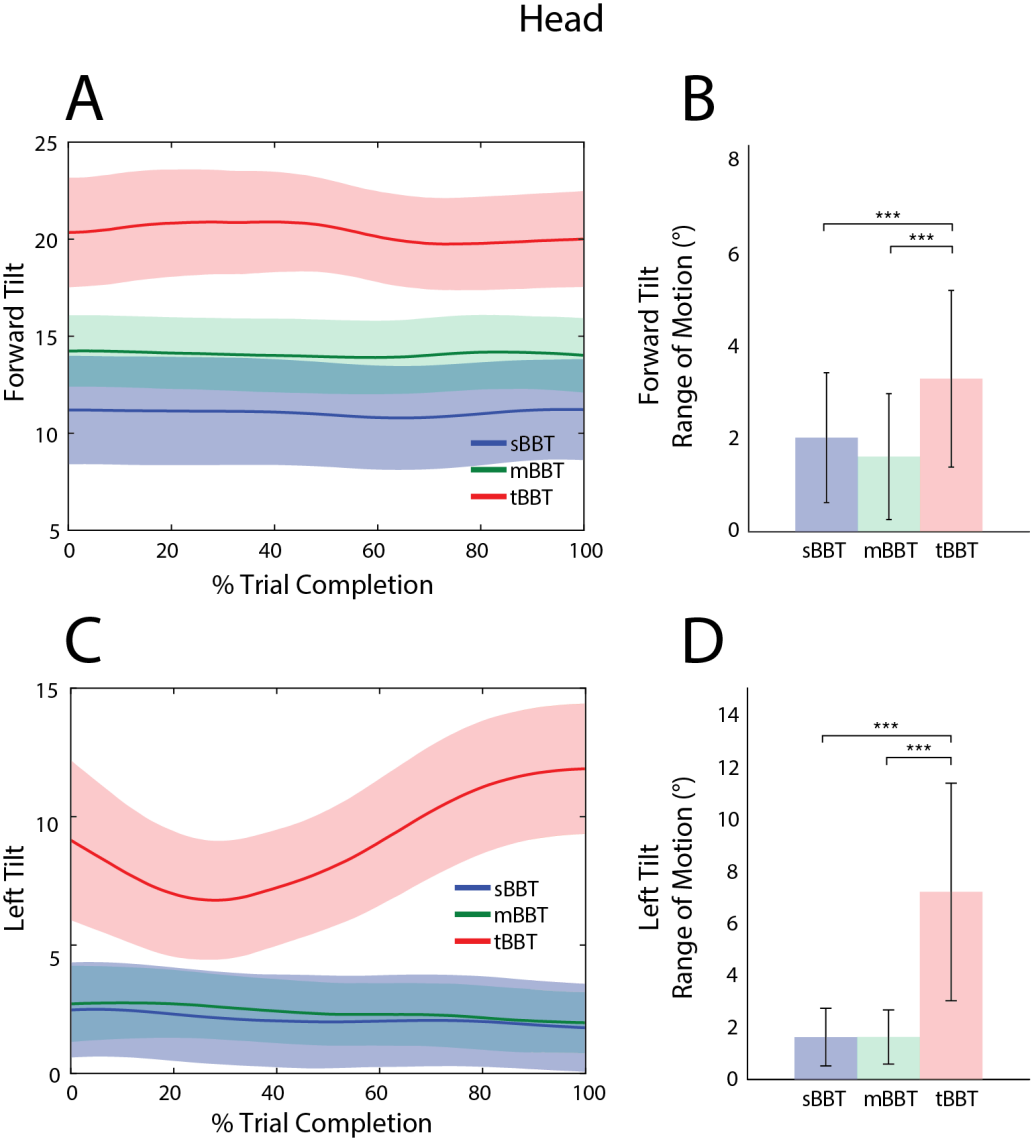
Kinematic trajectories and RoM of the head. Kinematic trajectories and RoM of the head for the sBBT (blue), mBBT (green), and tBBT (red). (A) Forward tilt and (C) left tilt trajectories for each test. (B) Forward tilt RoM and (D) left tilt RoM for each test.

Angle trajectories and RoM for forward and left head tilt are shown in Fig 7 for subjects performing all BBTs. During performance of the tBBT, subjects tilted their heads forward about 20° for the duration of the task while subjects performing the sBBT and mBBT only required approximately 11° and 14° of forward head tilt, respectively (Fig 7A). The head forward tilt RoM required during the tBBT was statistically significantly higher than the other tests (Fig 7B). No significant differences between the sBBT and mBBT forward head tilt RoM were found.

The left tilt angle trajectories of the head for the sBBT and mBBT were similar, with no significant differences between the peak angles and RoM (Fig 7C and 7D). Subjects maintained a fairly neutral head position in the coronal plane, with the left tilt head angle averaging 3° for all subjects during these tasks. The tBBT task required significantly different head motion in this plane as evidenced by the significantly greater ROM and peak angles. As subjects performed the tBBT, the head tilted to the left to a greater degree during transport and placement of the blocks on the opposite side of the partition, reaching a peak angle of approximately 13° at trial end (Fig 7C).

### Center of Pressure (CoP)

Kinetic data acquired through two ground force plates were analyzed to calculate the combined CoP. Several parameters of the CoP were determined and compared to assess differences in movement during performance of the three BBT variants. Raw force plate data can be found in the S1 File.

**Fig 8:**
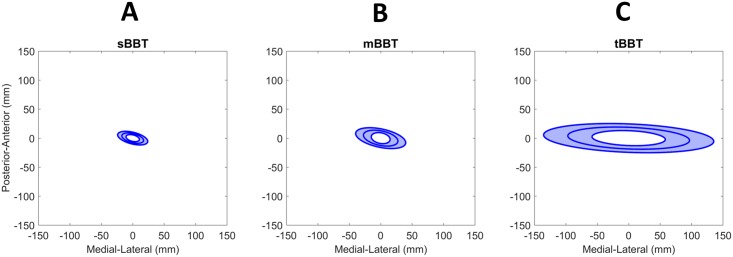
Center of Pressure. Average CoP 95% confidence ellipse for subjects (n = 16) performing the (A) sBBT, (B) mBBT, and (C) tBBT. The y-axis and x-axis show the zero-mean CoP in the anterior-posterior and medial-lateral directions, respectively.

The average 95% confidence ellipse for the CoP of subjects (n = 16) performing each BBT task is shown in Fig 8. Significant differences between BBTs were found (p < 0.05). Post-hoc comparisons showed that the CoP area during tBBT (mean 50, SD 29 cm^2^) was significantly greater than the CoP area during mBBT (mean 9.7, SD 5.9 cm^2^) and sBBT (mean 4.3, SD 2 cm^2^).

Several other CoP parameters for each test are shown in Table 1. For all parameters, the Friedman test showed significant differences between all three BBTs (p<0.05). Post-hoc comparisons revealed which tests differed significantly. CoP velocity significantly differed across all tests: tBBT required the greatest average velocity and sBBT required the lowest (p < 0.05). Acceleration was not significantly different between the tBBT and mBBT, but the accelerations of the CoP during both tests were significantly greater than those required during the sBBT. The total distance traveled during transport of the 16 blocks was significantly greater for the tBBT compared to the mBBT and sBBT.

**Table 1:** CoP parameters for each BBT.

CoP Parameter	Mean (SD)			p-value
	sBBT	mBBT	tBBT	
Velocity (mm/s)	29.1 (11.1)	46.2 (21.3)	105.2 (56.8)	< 0.001*
Acceleration (mm/s^2^)	3.8 (1.7)	5.6 (3.2)	8.8 (5.4)	< 0.001*
Distance Traveled (mm)	435.1 (167)	536.8 (193.8)	2420.9 (1203.4)	< 0.001*
AP range (mm)	22.9 (8.5)	32.6 (11.1)	48.8 (11.8)	< 0.001*
ML range (mm)	39.9 (15.4)	62.9 (29.8)	170 (64.2)	< 0.001*
Ellipse ϕ (rad)	2.9 (0.4)	2.9 (0.3)	3.1 (0.1)	< 0.001*

* indicates p values are significant at p < 0.05

## Discussion

In this study, we introduce the targeted BBT (tBBT), a novel functional outcome measure that can be used to assess the functional abilities of individuals with upper limb impairments and disabilities. Performance and movement data of able-bodied subjects assessed on this novel metric as well as on the standard BBT (sBBT) and modified BBT (mBBT) were collected and analyzed. Angle trajectories and RoM of five key joints in the upper body, as well as simultaneous CoP data, were calculated and compared across the three tests.

For all kinematic trajectories except wrist flexion and elbow flexion, no significant differences were found between elicited movements during the sBBT and mBBT. This is supported by qualitative similarities in the kinematic trajectories in Figs 3–7, as well as quantitative evidence showing that the RoM and peak angles achieved during subjects’ performance of the sBBT and mBBT did not significantly differ. For most of the kinematic parameters evaluated in this study, however, the tBBT required significantly greater RoM and peak angles. These differences and their implications for the ecological validity of the tBBT are discussed further below.

The required ROM and peak angles in the shoulder and thorax were generally much larger in the tBBT compared to the other tests. Some of these peak angles and RoM values were close to those found in studies of the movements of able-bodied individuals performing activities of daily living [12, 13, 15]. For example, shoulder abduction ROM during the tBBT was found to be 19°, close to published results for opening a door (15°) [15], pouring liquid from one container to another (20°) [13], and transferring a weighted object (22°) [12, 13]. This result suggests that the tBBT task elicits movements that are more similar to those executed in everyday activities, and that it may therefore provide a more realistic idea of how well an upper limb amputee operating a prosthetic limb, or a person with an upper limb disability, will function in the real world. The results also showed that head forward tilt and left tilt ROM as well as peak angle required during the tBBT were statistically significantly higher than during the other tests (Fig 7). Head angle rotated towards the block target destination could be an indicator of higher cognitive load or attentional demand imposed by the requirement for ordered selection and controlled placement of the block. The differences in the trajectories of the thorax and head angles could also be an indicator of the different movement planning strategies employed by subjects for each test. The end goal of the tBBT is to control the placement of the block on the opposite side of the partition, while the end goal for both the sBBT and mBBT is to toss the block over the partition. During the first 10–25% of the trial (i.e. as the hand approaches the block for pickup), subjects performing the sBBT and mBBT were fairly upright with approximately 1° of lateral thorax flexion and remained upright during the entire task whereas subjects performing the tBBT were adjusting the thorax position between 7° and 5°, presumably to prepare for the controlled transport and release of the block (Fig 6B).

While most kinematic parameters showed greater RoM and high peak angles during tBBT, a different result was seen with the wrist flexion/extension and internal rotation. Fig 3A indicates that the tBBT required less ROM in the wrist than the mBBT and sBBT. Taking into account the approach and goals for each task, this result was not unexpected. Given the random organization of the blocks on the right side of the partition in the sBBT, more wrist flexion was needed to forage through the block pile to find a block that could be picked up. This is supported by the large standard deviation in wrist flexion/extension during the first 40% of the sBBT trials (Fig 3A). Also, during the release of the blocks in the sBBT and mBBT, the subjects generally tossed the blocks over the partition, whereas the tBBT required controlled transport of the blocks that was apparently achieved using more upper body movement than wrist movement. This observation is highly relevant to our understanding of everyday object transport tasks.

Several parameters were extracted from force plate data to characterize movement and postural control [23, 27]. Statistical tests showed that the CoP areas of subjects performing the tBBT task were significantly greater than those of subjects performing mBBT and sBBT. The CoP area observed for the tBBT is consistent with previously reported CoP areas for subjects performing table-top tasks (e.g. moving objects from side to side, pegboard transfer task), and indicates that this test elicits movements more commonly seen in everyday life [23, 28].

Variability in the CoP ranges and area tended to increase when subjects performed the tBBT task compared to the sBBT and mBBT, as did performance scores (Fig 2). This variability could be due to \differences in the way subjects interpreted instructions, specifically whether they chose to optimize for accuracy (minimizing block placement errors) or speed. Variability could be reduced by making the test and instructions more explicit in emphasizing one goal over the other, or modifications to the test could be made to reduce the cognitive burden on placement of the blocks and allow the subject to focus more on the speed. Further development of the tBBT may include incorporation of a slightly raised frame with 16 square holes on the opposite side of the partition to make accuracy more consistent across subjects.

## Supporting information

S1 File Normal data. Raw kinematic and force plate data for each subject performing the sBBT, mBBT, and tBBT.
(XLS)
